# Sputtering of Electrospun Polymer-Based Nanofibers for Biomedical Applications: A Perspective

**DOI:** 10.3390/nano9010077

**Published:** 2019-01-08

**Authors:** Hana Kadavil, Moustafa Zagho, Ahmed Elzatahry, Talal Altahtamouni

**Affiliations:** Materials Science and Technology Program, College of Arts and Sciences, Qatar University, P.O. Box 2713, Doha, Qatar; hk1002981@student.qu.edu.qa (H.K.); mmsalah@qu.edu.qa (M.Z.); aelzatahry@qu.edu.qa (A.E.)

**Keywords:** electrospinning, sputtering, drug delivery, wound dressing, biocompatibility, tissue engineering

## Abstract

Electrospinning has gained wide attention recently in biomedical applications. Electrospun biocompatible scaffolds are well-known for biomedical applications such as drug delivery, wound dressing, and tissue engineering applications. In this review, the synthesis of polymer-based fiber composites using an electrospinning technique is discussed. Formerly, metal particles were then deposited on the surface of electrospun fibers using sputtering technology. Key nanometals for biomedical applications including silver and copper nanoparticles are discussed throughout this review. The formulated scaffolds were found to be suitable candidates for biomedical uses such as antibacterial coatings, surface modification for improving biocompatibility, and tissue engineering. This review briefly mentions the characteristics of the nanostructures while focusing on how nanostructures hold potential for a wide range of biomedical applications.

## 1. Introduction

In the last 20 years, the emergence of nanotechnology has drawn much attention to the electrospinning process. This process is used for the preparation of polymer Nano-micro fibers, and it has great importance in the biomedical industry due to its cost-effectiveness, scalability, versatility, and simplicity. The process was developed in 1901 by JF Cooley and WJ Morton, but had slower development over the subsequent 100 years. In more recent years, Reneker [1] developed fiber preparation from an organic polymer, which created a new field of science for the formulation of fiber diameter ranging between 1 × 10^−9^ and 1 × 10^−6^ m.

**Figure 1:**
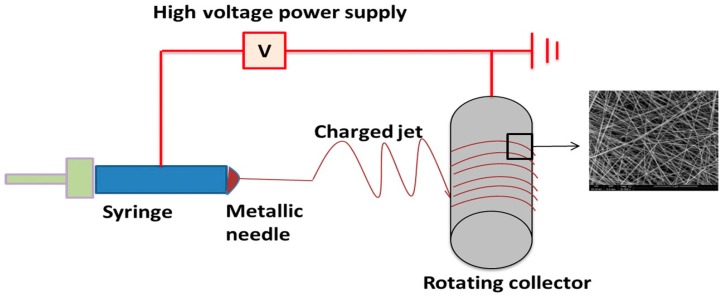
Schematic representation of electrospinning apparatus with a rotating collector. Reproduced with permission from [Coatings]. Copyright MDPI, 2014.

Electrospinning devices include four main components (Figure 1): high power supply, syringe pump, syringe needle with solutions, and a collector for fiber deposition. The electric field is applied between the needle and the collector, where the positive electrode is connected to the needle and the negative electrode to the collector. Hence, when voltage is applied, the repulsive charge accumulates at the tip of the needle, which is shaped in the form of a hemisphere [2]. When the repulsive charge overcomes the surface tension, it leads to the formation of a Taylor cone. This directs the polymer solution to the negative electrode, as the collector, and produces fibers. The solvent from the polymer solution is evaporated, and the polymer solution is deposited on the collector as dry fibers ranging in size from nanometers to micrometers [3].

### 1.1. Electrospinning Parameters

There are many parameters controlling the electrospinning process. These include solution parameters and processing parameters. The solution parameters include the concentration of the polymer solution, molecular weight, viscosity, surface tension, solvents, and conductivity/surface charge density. Additionally, processing parameters such as voltage, collector/needle distance, flow rate and the diameter of the syringe, along with ambient parameters like humidity and temperature, also play a major role in the fabrication of nanofibers for electrospinning process [4]. Customized electrospun fibers are produced by changing the parameters above [5]. Understanding these parameters is necessary in order to optimize the fiber structure.

#### 1.1.1. Solution Parameters

The solution parameters are important criteria for the production of uniform fibers. Of these, concentration is the most powerful tool for governing the morphology of the electrospun nanofibers. Electrospraying occurs when the concentration of the solution is too low, resulting in the development of micro- or nanoparticles [6]. However, smooth fibers are established when the concentration is increased [7]. However, increase in the concentration beyond a certain point will lead to the formation of a ribbon-like morphology. Simultaneously, higher molecular weight also leads to the ribbon-like structure for the nanofibers, even at a lower concentration of fibers [8]. Surface tension is also an important parameter, and depends on solvent composition. It has been noted that a reduction in the surface tension of the solution can lead to smooth fibers at a fixed concentration due to a smaller electric field being required to overcome the surface tension for the formation of uniform Taylor cones [9,10]. Concurrently, viscosity also performs a critical role in the electrospinning process. A lower-viscosity polymeric solution cannot be electrospun, but higher-viscosity solutions also cause problematic ejection jets in the solution. Therefore, viscosity adjustment is also an important factor for continuous and uniform fiber formation with continuous Taylor cones [11]. Additionally, conductivity also plays an important role in fiber formation. Conductivity depends on the type of polymers, solvents, and addition of salts. It has been found in the literature that, the increment in the conductivity leads to thinner and more uniform fiber formation [12]. All solution parameters are linked with one another [13]. It follows that solution optimization is an important step for the design of uniform and continuous fiber morphologies in the electrospinning process.

#### 1.1.2. Processing Parameters

Processing parameters are also an important factor in the electrospinning process. These include voltage, tip–collector distance, flow rate, type of collector, and electric field. Voltage influences the anatomical morphology of the nanofibers due to the dependency on fluid flow dynamics [14]. Many authors have found a non-linear relationship between fiber diameters and voltage [15]. The level of impact of voltage on the fiber diameter is known; however, polymer concentration and tip-to-collector distance play a more significant role than voltage [16]. Tip-to-collector distance has shown direct proportionality with fiber diameter due to solvent evaporation at larger distances exhibiting thinner fibers, and vice versa [17]. Furthermore, flow rate also plays a key role in fiber morphology. As the flow rate increases, the fiber diameter increases directly with thicker beads [13]. The type of collector also influences the fiber morphology. There are different types of collectors, including pins [13], girded bars, and rotating rods/wheels [18]. It has been noted that a smaller collection area shows a negative effect on fiber morphology with respect to the formation of beads [19]. The electric field plays a very important role in the electrospinning process. The electric field can be approximated as the voltage divided by the distance between the needle and the collector [20,21,22].

#### 1.1.3. Ambient Parameters

Environmental conditions also affect the morphology of the electrospun fibers. For instance, temperature, pressure, and humidity play a unique role. For example, electrospinning under high vacuum may lead to higher electric fields, and thus the formation of larger fiber diameters [23]. On the other hand, Supaphol et al. observed thinner fiber of polyamide-6 at 60 °C than at 30 °C [24], suggesting that an increase in temperature leads to a reduction in fiber diameter [24]. In addition, surface pores became apparent when electrospinning in an atmosphere with relative humidity higher than 30% [25].

The purpose of this review is to highlight the benefits of employing electrospun and sputtered electrospun polymer nanofibers in current biomedical applications connected to electrospinning and sputtering technologies. Furthermore, a novel approach is discussed for the processing of fibrous materials for different biomedical applications by combining electrospinning and sputtering technologies.

## 2. Biomedical Applications of Polymer-Based Electrospun Nanofibers

It is worth noting that polymer-based composites are widely used in a variety of different applications [26,27,28,29,30,31,32,33,34,35,36,37]. The fibers prepared by the electrospinning process have a high surface-to-volume ratio, adjustable porosity, tailored composition, and other favorable properties. To take advantage of this, a wide variety of polymers can be electrospun, including natural polymers, synthetic polymers, and biodegradable polymers. These micro/nanofibrous polymers have several advantages, including the fact that the fiber scaffold mimics the extracellular matrix, thereby enhancing cell adhesion, proliferation, migration, and differentiation. On the other hand, the pore size of electrospun membranes is too small to house cells inside the pores, and the cells spread out on the surface of the material [38]. This makes possible the release of biofactors such as drugs, proteins, and genes, as well as promoting nutrient and oxygen diffusion and waste removal. Also, the morphology of electrospun nanofibers—including core/shell, hollow, nanowire-micro tubers, and three-dimensional fiber scaffold morphologies—can be modified by changing the parameters of the electrospinning process. Thus, these beneficial factors make electrospun nanofibers suitable for biomedical applications such as drug delivery, tissue engineering, and wound healing. 

### 2.1. Drug Delivery

The idea of drug delivery emerged in the 1970s for the controlled release of drug for treatment [39]. The high surface area and porosity of polymer fibers has attracted great attention in recent years for use as a drug carrier. The use of the electrospinning technique can modify polymer fiber morphology and bulk properties. In this process, polymer nanofibers loaded with drugs are synthesized for drug delivery. Drugs ranging from antibiotic and anticancer agents to proteins, aptamer, DNA [40] and RNA [41] have been incorporated into nanofibers. The release mechanism of drugs in polymer fibers can be altered by changing the type of drug loadings employed, which include co-axial electrospinning, emulsion electrospinning, multiple layers, blended electrospinning, co-electrospinning, etc. However, co-axial electrospinning and multi-layered electrospun fibers have shown great application in drug delivery due to a sustained release of the drug, rather than an initial burst release of drug from the fiber scaffolds. Therefore, recent advancement in the field of electrospinning for drug delivery will be discussed in the proceeding paragraphs. The discussion of electrospinning in drug delivery will be broken down into categories of drug loading types, drug loading materials, types of drugs, and mathematical modeling of drug delivery systems, as explained in detail below.

#### 2.1.1. Drug Loading Types

**Figure 2:**
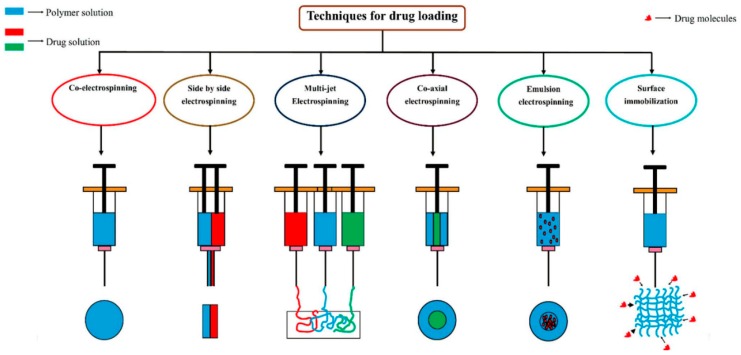
Schematic representation of different types of electrospinning processes. Reproduced with permission from [42]. Royal Society of Chemistry, 2015.

Electrospinning has different drug loading types, which determine the diverse structure and drug release kinetics. The drug loading procedure in electrospinning can be executed in different ways, including co-electrospinning, multi electrospinning, side-by-side electrospinning, co-axial, surface immobilization and emulsion electrospinning (Figure 2). In co-electrospinning, the drug molecules are mixed with the polymer solution before electrospinning. These electrospun fibers provide a uniform distribution of drugs/biomolecules and high drug/biomolecule loading. However, the biomolecule properties can be negatively affected when they are directly exposed to high voltage. On the other hand, blend electrospinning and side-by-side electrospinning help to solve the issue of drug and molecule solubility in common solvents. Moreover, multi-jets with more than two spinnerets represent a way of protecting the bioactivity of the drug. In addition, surface immobilization is another method, in which drug molecules are covalently bonded with the scaffolds via chemical or physical immobilization methods. In these chemical methods, the surfaces of the nanofibers are changed by introducing amines, carboxyl, hydroxyl or thiol; the physical methods involve the incorporation of Van der Waals, electrostatic and hydrophobic interactions. These methods of immobilization retain biomolecular activity, but the drug molecules in all electrospinning processes exhibit burst release kinetics. To overcome this, co-axial and emulsion electrospinning processes were introduced. Co-axial and emulsion electrospinning processes have been gaining increasing interest recently due to their promising ability to shield the biomolecules with the core and to minimize the drawback of the initial burst. They provide sustained release of the drug by minimizing the initial burst release by controlling the thickness and composition of the shells. The core/shell structure is generated utilizing a single-nozzle electrospinning unit employing emulsion input, commonly named emulsion electrospinning [42]. Another means of drug loading for sustained release of a drug is layer by layer, via the addition of drug in between the electrospun scaffolds; controlled release is promoted by the shield provided by the layer of polymer scaffolds. Therefore, co-axial electrospinning and multilayer electrospinning drug loading types provide a more sustained and controlled release of the drug.

#### 2.1.2. Multiple Layered Fiber Mats

**Figure 3:**
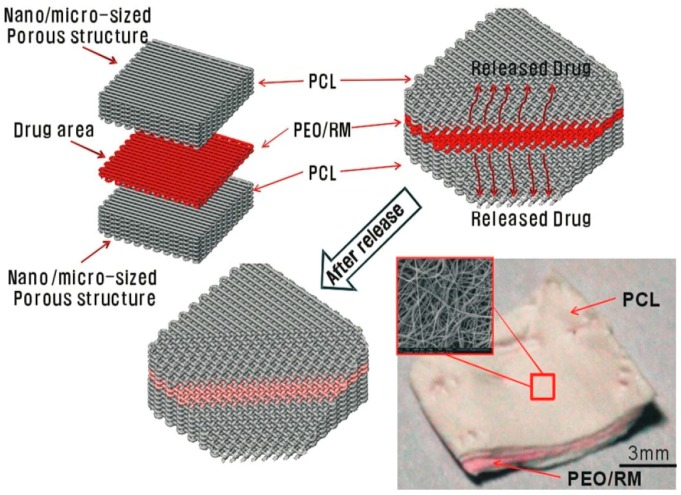
Schematic of the new drug release system consisting of two different electrospun mats. The inner and outer parts of the mat were PEO/rhodamine and PCL fibers, respectively. The red color shows that the rhodamine was well embedded in the PEO/rhodamine mat. Reproduced with permission from [44]. Springer Nature, 2010.

Multilayered fiber mats provide controlled release of a drug through the layer-by-layer stacking of a nanofiber sandwich, with drug loaded in between. This type of design is straightforward, easily controllable, and with a simple fabrication process as compared to the core/shell design. The drug release mechanism of multilayer fibrous mats can be controlled by adjusting the thickness of the outer layer, the amount of drug loaded, the porosity of the scaffolds, etc. The design of a core/shell structure is a complicated process, in one sense, due to the diverse electrical and rheological properties, such as conductivity and surface tension, of the core and shell polymer materials [43]. Hence, due to the difficulty of fabricating a core/shell design, electrospun fiber mats are not able to achieve sustained repeatability; additionally, controlled release of drug from the structure is difficult to be investigated efficiently. GeunHyung Kim [44] prepared polycaprolactone (PCL)-PEO-PCL layered fiber mats, and drug delivery was examined with various thicknesses of PCL outer layer. It was shown that burst release can be avoided by increasing the thickness of the PCL layer, as well as by incorporating antimicrobial peptide HPA3NT3, which does not lose its biological activities (Figure 3).

On the other hand, sustained release of the drug haloperidol was investigated by changing the hydrophobicity of the scaffolds. Therein, polyvinyl alcohol (PVA)-methylated b-cyclodextrin was incorporated with PLA and PLGA. The addition of b-cyclodextrin reduced the fiber degradation rate of PVA [45]. It was noted that when the hydrophobicity of the scaffold was increased, the release of the hydrophilic drug was sustained in a controlled manner, while polyester polymers released the drug by means of hydrolysis. The blending of the hydrophobic and hydrophilic drug will minimize the toxicity caused by the burst release of the drug. This type of combination can be applied for hydrophobic and heat-sensitive drugs, due to the simplicity of the process.

The drug delivery of ibuprofen from sandwich-layered fiber mats was studied, and its mathematical modeling was elaborated by using applying the power law, the Higuchi equation, and Fick’s second law [46]. The mathematical modeling suggests that the thickness of the fiber mats have a greater impact on drug delivery than the concentration of the loaded drug. Here, PLA was successfully electrospun by incorporating ibuprofen drug in between the two layers of PLA. Finally, according to the type of treatment, the drug loading can be changed by altering the thickness of the layers for controlled release of the drug. Dave Wei-Chih Chen et al. [47] studied drug delivery of vancomycin, gentamicin, and lidocaine for wound-healing applications. In their research, they successfully mixed PLGA/collagen on the outer layer and PLGA loaded with a drug in the middle layer. The drugs vancomycin and gentamicin were released in high concentrations from the biodegradable polymer scaffold. However, lidocaine showed a release time of up to 3 weeks. The bioactivities of the drug were shown to exhibit 40–100% efficiency, and it was concluded that this scaffold was suitable for boosting the wound-healing process in the initial stage of the wound. 

#### 2.1.3. Drug Loading Materials

Varieties of polymers can be electrospun into diverse designs for drug delivery applications, taking account of polymer–drug compatibility and their ability to be molded to fit a range of delivery routes. When designing an optimized drug delivery system, there are many polymer factors to be considered. For instance, biocompatibility, biodegradability, mechanical properties and hydrophilicity [48]. There are many polymer varieties, such as natural and synthetic polymers, that are used for designing drug delivery systems [49,50]. A diverse range of drugs have been loaded into delivery systems, including growth factors, DNA, proteins, inhibitors, and antibiotics [33,34,35]. 

Electrospinning processes can be applied to synthetic polymers easily and with great flexibility. However, synthetic polymers affect cell affinity due to their hydrophobic nature and the smooth surfaces of their cell recognition sites. On the other hand, natural polymers show enhanced biocompatibility, and some exhibit antibacterial properties and better clinical functionality.

The group of natural polymers includes cellulose, chitosan, chitin, dextrose, collagen, silk, gelatin, etc. [51]. Lee et al. investigated the features of different polysaccharides upon electrospinning, as well as their biomedical applications, such as drug delivery, wound dressings and enzyme immobilization [52]. The studied polysaccharidses included cellulose, chitosan, alginate, chitin, starch, hyaluronic acid, dextran, and heparin. Chitosan polymer had anticancer properties due to its polycationic nature.

The quartininized form of chitosan is well known for its improved in vitro anticancer ability against Hep3B, HeLa and SW480 cells [53]. However, natural polymers lack mechanical strength, and have a relatively sudden degradation rate due to their hydrophilic nature, inhibiting their use in long-term drug delivery process. In addition, the disadvantages of immunogenicity, batch-to-batch differences, limited availability, expensive production and vulnerability to cross-contamination all limit their clinical application [54].

On the other hand, the limitations of natural polymers could be overcome in application through the use of synthetic polymers, which mainly include biodegradable polymers such as PCL, PVA, polylactic acid (PLA) and Polyglycolic acid (PLGA). These synthetic polymers can be degraded via enzymolysis or hydrolysis. These materials are therefore of great importance in drug delivery, as drug delivery for the tissue regeneration process can take time; also, tissue regeneration can occur [55]. The rate of degradation depends on the sustained release of the drug, such that the degradation rate can be controlled by changing parameters such as the ratio of amorphous to crystalline segments of polymers and polymer blend compositions [41,42]. Synthetic polymers have many advantages in comparison to natural polymers, as they are inexpensive, have excellent mechanical properties and tunable degradation, as well as exhibiting great durability. However, they also have disadvantages, such as lack of cell-specific recognition sites due to their smooth and hydrophobic surfaces.

The production of novel composite fibers through the combination of synthetic and natural polymers could reduce the disadvantages [56,57]. The combination of natural and synthetic polymers would help in the formation of a fiber that was the same as the extracellular matrix, with outstanding mechanical properties and adjustable biodegradability. For example, PLGA-gelatin was fabricated by blending electrospinning for the drug delivery of fenbufen (FBF) [58]. These blended scaffolds have optimized mechanical properties, degradation rates and bioactivites. However, the drug release profile could be controlled by increasing the volume of PLGA in the blend. This would make the scaffolds more hydrophobic, resulting in a slower degradation rate. In another paper, composite scaffolds were prepared through a combination of PCL-gelatin, resulting, because PCL is a hydrophobic polymer, in tunable hydrophobicity, degradation rate, and mechanical properties.

Simultaneously, gelatin provided cellular attachment and adhesion of bone marrow derived from human mesenchymal stem cells (hMSCs). Thus, these types of tunable properties could result in promising scaffolds for drug delivery applications and tissue engineering systems [59]. While designing a system for the sustained release of a drug, many factors contribute to the efficient release of drug from the polymer scaffolds. These elements include the degradation and wettability of the polymer scaffolds, the type of drug and the drug loading type.

For the sustained release of the drug, the most important factor is the drug loading type. There are many types of loading, including co-axial electrospinning and multilayer electrospinning, which shows a controlled release of the drug over a longer term. The sustained release of the drug depends on the following factors in coaxial electrospinning: the thickness of the shell layer, porosity, degradation rate of the shell fiber, the hydrophobicity of the scaffolds, etc. On the other hand, in multilayered electrospinning, the drug release kinetics depends on the scaffold porosity, the thickness of the outer layer, the hydrophilicity of the scaffold, etc. The following sections describe co-axial electrospinning and multilayered electrospun scaffolds prepared by PVA hydrophilic and PCL hydrophobic polymers incroporating various drugs.

##### Polycaprolactone (PCL)

Polycaprolactone (PCL) is a hydrophobic polyester polymer widely studied in electrospinning. PCL has wide biomedical applications due to its biocompatibility, biodegradability, mechanical properties, non-toxicity, low cost, and low melting point. Commercially available PCL has a molecular weight ranging between 3000 and 85,000 g/mol. PCL is a hydrophobic molecule. Hence, it dissolves in solvents like chloroform, acetone, acetic acid, dichloromethane, toluene, methanol, benzene and tetrachloride [10]. The properties of PCL, including biodegradability, cytotoxicity and degradation rate, have been studied elaborately with respect to short- and long-duration implants [60,61]. Degradation of PCL is non-enzymatic, and occurs by means of hydrolysis. PCL fibers have been widely studied as a drug carrier in drug delivery.

##### Polyvinyl Alcohol (PVA)

Polyvinyl alcohol (PVA) is a semicrystalline hydrophilic polymer that is easily soluble in water. The solubility in water gives PVA wide applicability in drug delivery [62,63]. PVA is a biocompatible, biodegradable and easily electrospinnable polymer. PVA has been used as a sacrificing template for the preparation of non-electrospinnable polymers. However, PVA alone cannot be used for drug delivery due to its water solubility. PVA was fused with chitosan to improve its biocompatibility and cell attachment [47,48]. Gelatin electrospun with PVA was used as a template for improved gelatin fibers [64]. However, PVA has poor mechanical properties. Therefore, many scientists have tried to study composite materials that might enhance the mechanical properties of PVA [64]. To avoid the burst release of drugs, Zupančič et al. synthesized core/shell nanofibers with a poly(methylmethacrylate) (PMMA) shell and a monolithic PVA core, or novel core/shell nanofibers with a blended PMMA/PVA core loaded with ciprofloxacin hydrochloride (CIP) [65]. The combination of PVA with PCL polymer has gained much attention recently, because the addition of PCL might enhance its mechanical characteristics. Therefore, the study of PCL/PVA as a multilayer scaffolds for the sustained and controlled release of drug is described below.

##### Combined PCL/PVA

Multilayered structures have gained much attention due to their versatility and controlled release of drugs. The drug was studied as a middle layer, and the outer layer requires the controlled release of antibiotics. For instance, Liu et al. [66] prepared a novel scaffold by integrating a 3D bioprinting platform and electrospinning in order to study multiple drug delivery. Here, PVA blended with gentamicin sulfate and co-axial PVA-DFO/PCL was fused layer by layer to form a 3D scaffold for osteointegration and sustained drug release. Burst release was noted for gentamicin sulfate, but the sustained and controlled release of DFO due to the presence of a vertical gradient of sodium alginate/gelatin in the scaffold give the DFO a gradient mode of release. Therefore, a combination of 3D bioprinting and electrospinning can be used to prepare functional gradient scaffolds. In another study, the release of the model drugs tetracycline hydrochloride (TC-HCL) and phenytoin sodium from PVA-PCL-PVA multilayered electrospun nanofibers was reported [67]. Hydrophilic and hydrophobic polymers were prepared layer by layer by incorporating multiple drugs such as PHT-Na with OVA and TC-HCL with PCL, respectively. 87% of the TC-HCL was released from a single fiber, and only 47% was released from the multilayer scaffolds. The release kinetics mechanism was Fickian diffusion, and the release profile corresponded to the Korsmeyer-Peppas equation. These materials had great application in wound dressing mats. Multilayered electrospun fiber scaffolds have great importance in drug delivery.

#### 2.1.4. Types of Applied Drugs

##### Antibiotics and Antibacterial Agents

Antibiotics and antibacterial agents have been incorporated for the enhancement of scaffold properties. Ignatova et al. studied the use of several antibiotics in electrospun scaffolds and their application for wound dressings [68]. The antibiotics included tetracycline hydrochloride, ciprofloxacin, moxifloxacin, levofloxacin and antibacterial agents (for example, 8-hydroquioline derivatives, benzalkonium chloride, itraconazole, fusidic acid, and silver nanoparticles (Ag NPs)). Gentamicin sulfate-loaded PLGA and gelatin were also studied for the continuous release of drugs [69]. The results showed that 70/30 PLGA/gelatin nanofiber scaffolds exhibited a gradual release of the drug over the first 15 h, rather than a burst release effect, indicating that this is a promising scaffold for wound healing applications. On the other hand, the drug release profile was studied for a polyethylene covinyl acetate and PLA blend scaffold in which tetracycline hydrochloride was the model drug [70]. The drug delivery release profile depends on the type of fiber and percentage of drug content. The 50/50 blend provided about 5% release of tetracycline hydrochloride within 5 h, with a regulated and smooth release thereafter. Additionally, 25 wt% exhibited a more rapid release than 5 wt% due to the surface segregation of tetracycline, which dissolves quickly.

Zhang et al. [71] electrospun nylon 6 nanofibers and electrosprayed TiO_2_ NPs onto them to fabricate highly porous photocatalytic TiO_2_ NP-decorated nanofibers with excellent antibacterial behaviors. Moreover, they also prepared solution-blown soy protein nanofibers decorated with Ag NPs. Another type of antibacterial electrospun nanofiber prepared from sodium alginate (SA)/PVA was discussed by Shalumon et al. [72]. Incorporating ZnO NPs increased the diameter of the prepared fibers. Antibacterial examinations confirmed that the processed mats displayed inhibition of both bacterial strains for all contents of ZnO NPs, and that the inhibition increased with an increase in the ZnO NP content [72].

Unnithan et al. prepared uniform nanofibers of polyurethane–dextran loaded with ciprofloxacin drug. The cell attachment and viability were improved after adding dextran to the polyurethane. The nanofibers displayed a good antibacterial activity for both Gram-positive and Gram-negative bacteria [73]. In addition, a biocompatible composite based on chitosan/collagen exhibited high liquid absorption and good antibacterial activity [74].

##### Anticancer Agents

Not only antibiotics, but also many other types of drugs, such as anticancer drugs, have been applied to the scaffolds of electrospun mats for chemotherapy. Diverse anticancer drugs, such as doxorubicin (Dox), paclitaxel (PTX), dichloroacetate and platinum complexes have been incorporated into the electrospun fibers for localized postoperative chemotherapy sessions. For instance, Xu et al. fabricated PEG-PLLA-loaded electrospun fibers via an water-in-oil emulsion method in which the aqueous phase was the hydrophilic drug, and the oily phase was the chloroform solution of PEG-PLLA [75]. The drug was well and uniformly dispersed in the PEG-PLLA fibers by using the electrospinning technique. In the same way, they successfully incorporated hydrophobic Paclitaxel (PTX) and DOX, which were simultaneously added to the nanofiber scaffolds via the emulsion electrospinning method; subsequently, multiple drug delivery was studied [76]. In contrast, Xe et al. prepared an electrospun scaffold of (30/70) PLA/PLGA blended fiber with the addition of cisplatin, and the results showed a 90% encapsulation efficiency; the sustained release of drug was noted for 75 days in the in vitro treatment of glioma [77].

##### Protein, DNA, RNA and Other Growth Factors

Over time, electrospinning has improved, thereby propagating many new and innovative ideas for biomedical applications. Blend electrospinning and co-axial electrospinning have been developed with the combination of protein, DNA, RNA and growth factors with the electrospun fiber mats for biomedical applications. The main challenge faced in this type of design is the loss in the bioactivity of the drug incorporated. Therefore, it is mandatory to optimize the material and electrospinning parameters for efficient results. Hence, the processes of blend electrospinning and co-axial electrospinning have drawn more interest towards this specific type of drug addition. Co-axial electrospinning is more efficient for protecting the bioactivity of the drug than blend electrospinning. Chew et al. encapsulated the human nerve growth factor, with BSA as a carrier, in polymers such as PCL and poly(ethyl ethylene phosphate) [78]. The results showed that there was a partial bioactive retention of the hNGF when the PC12 cell line was introduced to the scaffolds. There was a consistent release of hNGF for around three months, without burst release. The same group studied the release of small interfering RNA (snRNA) and transfection reagent (TKO) on electrospun fibers of copolymer caprolactone and ethyl ethylene phosphate (PCLEEP) [79]. The results showed a sustained release of siRNA for around 28 days. The copolymerization of ethyl ethylene phosphate with PCL led to improvements in the delivery rate of siRNA, as well as in gene knockdown efficiency, when compared to PCL alone. In co-axial electrospinning, the bioactive components are incorporated inside the core and are protected by the shell polymer. Hence, bioactivity can be protected from the electrospinning environment and the biological environment. Saraf and co-workers studied the incorporation of plasmid DNA (pDNA) into the core and shell polymers with non-viral gene carrier poly(ethleamine)-hyalouric acid (PEI-HA) [80]. The gene release was observed to last around 60 days by altering parameters such as the concentration of pDNA and the molecular weight of the core in order to control the transfer efficiency of the pDNA. The bioactivity of the drug could be controlled by the new design suggested by Mickova et al. [81]. They proposed the addition of liposomes to the core, which are able to hold the bioactive ingredients and protect their activity for effective action by shielding the lipid sphere from the electrospinning process.

#### 2.1.5. Mechanisms of Drug Release

**Figure 4:**
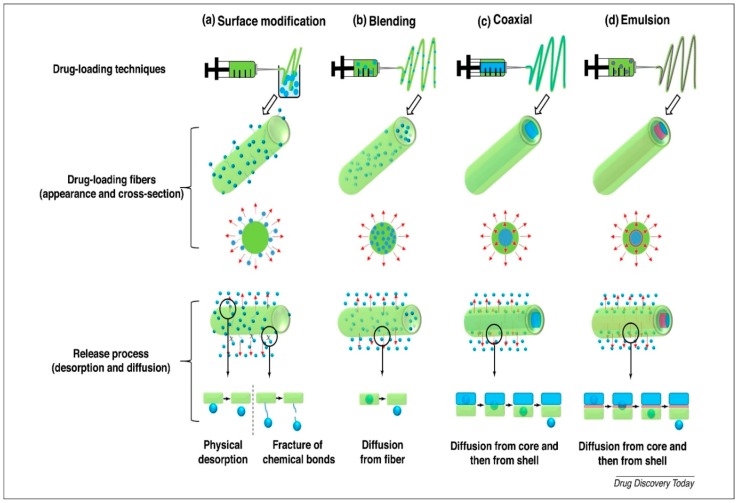
Drug loading and release (desorption and diffusion) from polymeric micro/nanofibers fabricated by (**a**) surface modification; (**b**) blending; (**c**) coaxial; and (**d**) emulsion electrospinning. The green color stands for the polymer, blue for drugs, and maroon for the surfactant. The red arrows represent the direction of the drug release. Reproduced with permission from [87]. Elsevier, 2017.

The release of drug from the scaffolds takes place via three mechanisms: desorption from the surface, diffusion through the fibers, and fiber degradation [82]. These three processes can occur simultaneously, which impacts the release kinetics throughout the entire process. Figure 4 provides a schematic representation of the drug release behavior of different types of drug loading. When the fiber is immersed in the aqueous media, the desorption mechanism occurs for drug on the surface of, as well as drug present inside of, the nanopores of the nanofibers [83]. Of these three mechanisms, desorption is undergone by drug on the surface of the polymer; therefore, burst release is observed. This burst release is due to the direct interaction of the medium with the polymer surface. Because burst release of a drug is not useful, surface modification is carried out, which is the main physical modification implemented for the controlled and sustained release of the drug to the environment. 

For example, Srikar et al. [84] embedded Rhodamine 610 chloride fluorescent dye in PCL/PMMA nanofibers to investigate the release of water-soluble compounds from electrospun polymer nanofibers. Furthermore, Gandhi et al. examined the release of serum albumin (BSA) and an anti-integrin antibody (Al) from electrospun PCL nanofibers [85]. The mechanism of release was observed to be dominated by desorption from the PCL surface. The two-stage desorption-controlled release of fluorescent dye Rhodamine B and vitamin B2 (riboflavin) from solution-blown and electrospun poly(ethylene terephthalate) (PET) nanofibers containing porogens was reported by Khansari et al. [86].

The second type of kinetics is the diffusion mechanism, whereby the concentration gradient causes the release of the drug into the medium. Herein, the diffusion process reduces the initial burst release and promotes a controlled and sustained release of the drug. Co-axial and emulsion electrospinning methods can exhibit this type of release kinetics. In emulsion electrospinning, drug droplets are well dispersed in the polymer solution before electrospinning [87]. A core/shell fibrous morphology comprises a core consisting of macromolecule aggregates in the aqueous phase, and a shell consisting of the polymers [87].

Finally, the third type of release mechanism is the degradation of the outer surface. For instance, using a low-degradability polymer as the shell will result in the sustained release of the drug due to the low degradation rate. In this sense, the mechanism of drug release kinetics can be optimized depending on the polymer incorporated and the type of electrospinning process. PCL is a low-biodegradability polymer; however, PVA is a highly biodegradable polymer. Therefore, a combination of these two polymers could provide a better drug release profile.

### 2.2. Tissue Engineering

Electrospinning techniques have received much consideration in tissue engineering. The electrospun fiber structure possesses many characters suitable for tissue engineering, including favorable mechanical properties, high surface-to-volume ratio, and adjustable porosity. Tissue engineering is an advanced field of science which merges applied engineering and bioscience in order to construct biomaterials that recover, sustain or improve the biological activities of injured tissues [88]. For efficient tissue engineering processes, three parameters need to be considered: seeding and attachments of cells, biomaterial scaffolds, and the addition of cell signaling factors. Of these, the biomaterial scaffold is a major parameter, and should mimic natural extracellular matrix with having sufficient mechanical properties, biocompatibility, biodegradability, high surface area, and high interpore connectivity. These criteria contribute to cell proliferation, differentiation, and migration. To this end, electrospun fibers can be prepared efficiently and cost-effectively to produce suitable candidate scaffolds for tissue engineering. Nowadays, several electrospun fiber mats are being prepared and studied for tissue engineering—with and without the addition of biological agents or growth factors—for wound healing, bond construction, and nerve tissue regeneration. 

#### 2.2.1. Skin Tissue Engineering/Wound Healing

In recent years, wound healing and skin tissue engineering have frequently been researched. Acute wounds normally heal in a very orderly and efficient process characterized by four distinct, but overlapping phases: hemostasis, inflammation, proliferation, and remodeling [89]. In the first phase, wound healing is stabilized by different cells, growth factors, and cytokines. The host cells and bacteria are removed by macrophages in the inflammation step. Then, fibroblasts migrate in to begin the proliferative phase and deposit new extracellular matrix. The new collagen matrix then becomes cross-linked and organized during the final remodeling phase. Therefore, effective wound dressing material is mandatory for proper treatment of wounds. Wound healing scaffolds should have good biocompatibility, mechanical properties and the capacity to prevent fluid evaporation from the injured site. Furthermore, it should provide a site for cell epithelization and inhibit infection [90].

Hence, the ability of cell attachment to electrospun fiber scaffolds plays an important role in the efficiency of engineered wound dressing scaffolds. The material and manufacturing process is important for the preparation of ideal wound dressings mats. Electrospinning is an ideal manufacturing process for wound dressing mats due to the above-mentioned advantages, such as biocompatibility, biodegradability, hydrophilic surface, porosity, and so on. Moreover, nanofiber scaffolds offer better clearing of exudates from the injured site, and manage both the loss of water from and the diffusion of oxygen in and out of the wound site [91]. There are natural (like collagen, gelatin, chitosan) and synthetic biodegradable polymers (PCL, PLGA, PGA, PLA, PVA, etc.) that can be molded together to form scaffolds. An electrospun scaffold is prepared in combinations of different natural and synthetic biodegradable polymers loaded with antibacterial and wound healing factors. The polymers can employ co-electrospinning, as well as blended, co-axial and multilayer electrospinning. Syed Mahdi Saeed [92] prepared a multilayered fiber mat loaded with curcumin as an active antibacterial component with novel PCL-PVA-PCL multilayered electrospun fibers. The results showed that multilayered PCL-PVA-curcumin-PCL illustrated better exudate absorbance than a pristine dressing at the incision. In the same vein, it indicates that 16% loaded curcumin displays antibacterial activity without killing the cell viability. Antibacterial properties can be built up in the scaffold with the addition of antibacterial agents or antibiotics. Silver is a well-known antibacterial agent, because it can mitigate the DNA replication of bacteria [93]. Khodkar and Ebrahimi [94] successfully prepared PVA/PCL core/shell fibers loaded with Ag NPs in the core for wound dressing applications. Fibers loaded with silver showed lower porosity, as well as an improved water vapor transmission rate (WVTR) and greater angle of contact. These scaffolds are suitable for long-term antibacterial activity (*Escherichia coli* and *Staphylococcus aureus*) because of the sustained and controlled release of the Ag NPs in the core/shell structure. On the other hand, PCL/PVA has been co-electrospun by loading silver sulfadiazine (SSD) as a drug for wound dressing mats [93]. PCL and PVA loaded with SSD were prepared successfully. The effect of different weight % of SSD on cell toxicity and mechanical and antibacterial properties was studied. Higher SSD concentrations were correlated with improved antibacterial ability, and cellular attachment, as well as proliferation, were observed. Fibronectin coatings can improve the biocompatibility of scaffolds loaded with SSD. Therefore, 5 wt% SSD-loaded co-electrospun PVA/PCL showed better antibacterial and reasonable cell proliferation and differentiation. Recently, Online et al. fabricated PVA merged with monodispersed Ag NPs and PCL loaded with Ascorbyl palmitate (AP) by dual-spinneret electrospinning [95]. The NIH-3T3 fibroblast cells were seeded on the scaffold mats, and it was shown that AP inhibits the toxic effects of Ag NPs on cell proliferation. It should also be noted that antibacterial tests confirmed the inhibition of gram-negative and gram-positive *Escherichia coli* (*E. coli*) and *Staphylococcus aureus* (*S. aureus*), respectively. Wound healing tests and histological observation concluded that this material provided a promising candidate for future biomedical applications [96]. Porosity and surface wettability are important parameters which determine the healing process. Xin Liu has electrospun PVA, PCL, PAN, and PVDF-HFP incorporating wool protein and Ag for wound dressing mats. Hydrophilic membranes have been shown to be an efficient remedy for wounds in comparison to hydrophobic membranes. Porosity, for the purpose of oxygen diffusion, also leads to an improved wound healing process. However, the wound healing process for diabetic ulcers is time-consuming due to the lack of efficient blood supply resulting from the higher amount of sugar in the blood. These processes lead to a long inflammatory stage, defective angiogenesis and blocked fibroblast proliferation.

Recently, Wang et al. [97] fabricated silk fibroin (SF)/GO nanofibers for wound healing applications. It was emphasized that graphene oxide enhanced the biocompatibility and antibacterial properties of SF composite nanofibers [97].

#### 2.2.2. Bone Tissue Engineering Applications

Bone is a strong rigid organ that plays an essential role in our body. It protects our vital interior organs, movements, manufactures white blood cells and red blood cells, and also stores minerals [98]. Bone extracellular matrix mainly consists of organic and inorganic components, such as collagen and hydroxyapatite (HAp). Incorporation of these components results in suitable scaffolds for bone tissue engineering applications. The architecture of the electrospun scaffolds, including microstructure, porosity and surface properties, plays an essential role in successful bone regeneration [99]. Electrospun fibers should offer better mechanical properties to support the structure, and provide space for osteochondral adhesion, proliferation, and differentiation. Hence, the development of an ideal scaffold for tissue regeneration could be achieved by using a porous ceramic material, lamellar material and a fiber matrix material for imrpoved biological and physical properties. Subramanian Uma Maheshwari developed a scaffold comprised of a polymer–ceramic combination in a PCL/PVA bilayer scaffold blended with HAp NPs [100]. (PVA-PCL)-HAp has an improved porosity of around 64%, as well as hydrophilicity of around 141%. Also, MTT assay studies with MG-63 osteoblast cells had better cell adhesion and proliferation, which indicates promise for application in tissue regeneration. However, the incorporation of growth factors (GFs) or drug to the scaffold is also crucial for enhancing the regrowth of broken bones. Many GFs, including bone morphogenetic protein-2 (BMP-2) and VEGF, have been added to electrospun scaffolds in order to achieve long-lasting sustained release of GFs to mimic the natural healing process. For instance, co-axial electrospun of collagen-PCL incorporating BMP-2 and dexamethasone (DEX) have shown a more controlled release of GFs, thereby encouraging the osteogenic expression of human mesenchymal stromal cells (hMSCs) [101]. In this design, the shell layer was loaded with DEX, and the core incorporated BMP-2. Dual drug release was exhibited, in which DEX showed a fast release. However, BMP-2 demonstrated a sustained release over 22 days. This scaffold provides an efficient healing process, as well as osteogeneration. 

On the other hand, incorporation of stem cells into the biomaterials is also a novel approach for tissue regeneration of the cells. For instance, Abbas Shafie has studied, in vitro and in vivo, cartilage tissue regeneration from rabbit bone marrow mesenchymal stem cells (BM-MSC) seeded on the electrospun scaffold of PVA/PCL nanofibers [102]. In vitro, the MTT assay showed that the scaffolds supported the chondrogenic differentiation of MSC. In vivo, the scaffold with and without MSC loading was implanted in rabbit full-thickness cartilage defects. To study cartilage regeneration, histological and semi-quantitative grading was executed. The results showed that scaffold seeded with MSC enhanced the healing process in comparison to non-seeded scaffolds. These results indicate that PVA/PCL scaffold seeded with MSC is suitable for grafts for articular cartilage repair.

Recently, the effect of polyacrylonitrile/MoS_2_ nanofibers on the growth behavior of bone marrow mesenchymal stem cells (BMSCs) was discussed by Wu et al. [103]. The nanofibers were realized to enhance the contact of BMSCs with each other, to enhance cellular behavior, and also to provide positive promotion of regulation of cellular proliferation [103]. In addition, for guided spinal fusion, an injectable and thermosensitive hydrogel made of collagen/n-HA/BMP-2@PCEC/PECE enclosed in poly(d,l-lactide) (PDLLA) nanofibrous membranes was made by Qu et al. [104]. This system restricted the escape factor in order to maintain osteogenesis in the desired position [104].

#### 2.2.3. Skeletal Muscle Regeneration

Skeletal muscle makes up around 40% of the human body. Skeletal muscle is made of various fibers, with diameters ranging from 10 to 80 µm [105]. These fibers are unidirectional and produce an enormous amount of force during contraction [106]. If a muscle cell gets injured or wounded, it will not be possible to contract, and satellite cells are switched on in order to perform their muscle cell regeneration activities. However, this healing process can create scar tissue and block muscle function [106]. Many efforts have been made to study the initial steps of muscle regeneration, such as autologous muscle transplant, satellite cells, exogenousmyogenic cells, and myoblasts, but these methods have met with limited success [107]. Therefore, long-term denervation and severe injuries can lead to the loss of skeletal muscle function.

Muscle tissue engineering materials require better contraction ability and mechanical properties [108]. Muscle cell adhesion and proliferation have been studied using both mechanical properties and electric stimulus in cell culture. Mckeon-Fischer K D prepared co-axial electrospun fibers with a PCL core and a modified outser shell layer comprising multiwalled carbon nanotubes (MWCNT) and a blend of (83/17, 60/40, 50/50, and 40/60) poly(acrylic acid/poly(vinyl alcohol) (PAA/PVA) [109]. All four components were electrically conductive, although the scaffold was not actuated when an electric field was applied. The best results occurred at 20 V. MTA assay in soleus and vastus lateralis (VL) muscles extracted from rats showed that 0%, 0.14% and 0.7% concentrations of MWCNT in the scaffold were non-toxic for cells over a four-week period. Based on the different percentages of blend solutions, 40/60 PAA/PVA in the outer layer illustrated a higher number cells than other scaffolds. The scaffold has tensile properties that are higher than those of skeletal muscle. Further modification of these scaffolds for contraction, rather than bending, could lead to promising scaffolds for artificial muscle applications.

#### 2.2.4. Nerve Tissue Engineering

Electro-conducting polymers such as polypyrrole (PPy), polyaniline (PANI), polythiophene (PT), poly(3,4-ethylene dioxythiophene) (PEDOT)) show attractive electrical and optical phenomena. Thus, they have been researched in the past few decades for various applications such as microelectronics, actuators and polymer batteries [110]. Electro-conducting polymers that possess the advantages of biocompatibility and good conductivity can be applied as biosensors and tissue engineering scaffolds [111]. Electrospun electro-conductive polymers are an excellent tool for electrically stimulating neurons and for nerve tissue engineering, as well as for application in neural prostheses for therapeutical function [88,89,112]. Schmidt et al. first studied PC12 cells using the polypyrrole (PPy) electroconductive polymer, recognizing the growth of PC12 cells on the PPy thin film, they enhanced the neurite outgrowth from the cells; these results suggest significant application of these type of scaffolds for nerve tissue regeneration [113]. Many studies have proposed the improvement of the electro-conducting polymer for nerve tissue regeneration applications by adding cell adhesive [114], neurotrophins [115], and topographical features [116]. Jae Y Lee prepared electrospun nanofibers coated with the conductive polymer PPy for nerve tissue engineering applications [117]. PPy-PLGA showed improved growth of rat pheochromocytoma 12 PC12 cells and hippocampal neurons compared to non-coated PLGA as a control. This suggests that PPy-PLGA could be used for nerve tissue engineering applications. Simultaneously, electrical stimulus studies on the scaffold indicated that a stimulus of 10 mV/cm improved the neurites such that they were 40–50% longer, as well as exhibiting 40–90% greater neuron formation, compared to the same scaffolds with no stimulus. Moreover, aligned scaffolds show greater neurite elongation and formation than randomly oriented PPy-PLGA fiber scaffolds. The good results for electric stimulus suggest that biocompatible polymers prepared by electrospinning have significant advantages in biomedical applications such as nerve tissue engineering.

## 3. Combination of Electrospinning and Sputtering Technologies for Biomedical Applications

### 3.1. Setup—Operating Principles of Sputtering Technology

**Figure 5:**
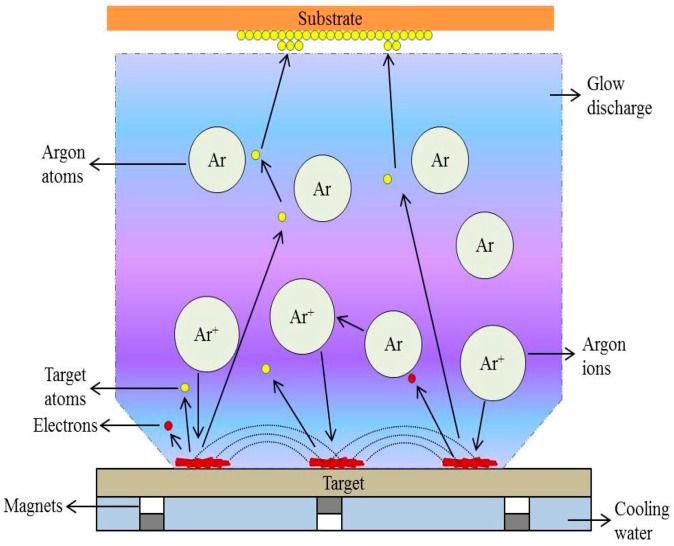
Schematic representation of sputtering technology [119].

Sputtering is the ejection of atoms by the bombardment of a liquid or solid target by energetic particles, typically ions [118]. These ejected atoms are then deposited on the substrate [118]. Figure 5 presents a schematic representation of sputtering technology. Argon gas is commonly used as a sputtering gas. The ejected fragments are accumulated on the substrate by adjusting the distance between the target and substrate. The number of atoms excited from the surface per incident ion is known as sputtering yield, ‘S’. The value of S depends on many parameters, including target material composition, experimental geometry, binding energy, and the properties of incident ions. In addition, there are also experimental parameters, such as voltage and current. In a conventional sputtering machine, the cathode is connected to the target and the anode is connected to the substrate, with the plasma in between them. 

**Figure 6:**
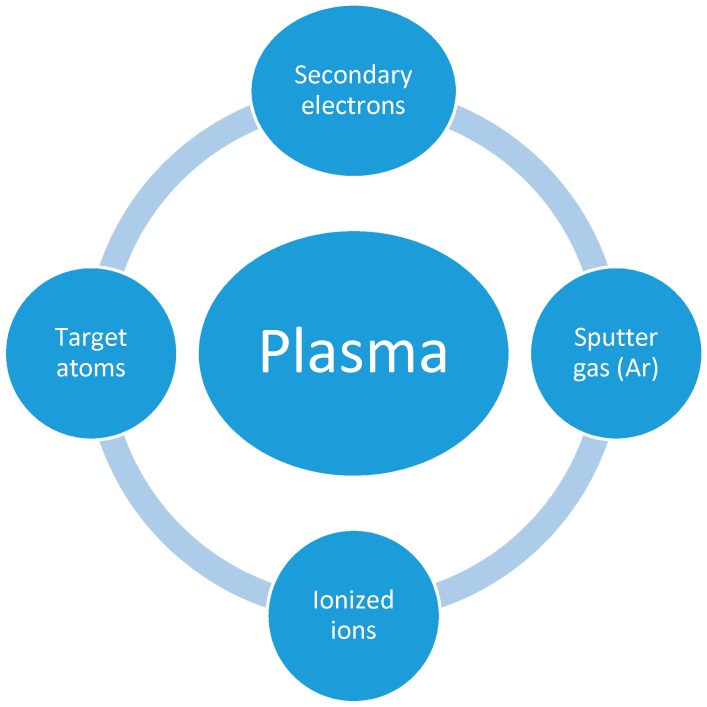
Factors affecting the plasma sputtering technique.

The direct current (DC) and radio frequency (RF) sputtering processes differ mainly with respect to the power supply installed. In the DC sputtering process, metals are only used as a target for coating. However, in the case of RF, insulators are used as a target for coating purposes. This is mainly achieved by providing the RF potential to the target. In practice, when insulators are used as the target, their charges are accumulated on the surface of the target after striking positive ions. This would make the target surface inaccessible for the further bombardment of ions. Therefore, to inhibit this process, both positive ions and electrons are bombarded directly onto the insulator target [120]. This is achieved by applying a RF power supply, which gives enough energy for the oscillating electron in the presence of an alternating field to originate ionizing collision, and a self-preserved discharge is maintained. However, the incorporation of magnetron sputtering into DC/RF sputtering could increase the sputtering yield by applying a strong magnetic field along the sputtering target to confine the plasma to the nearby target surface, on which the electric field $\vec{E}$ and the magnetic field $\vec{B}$ are used for electronic motion. Electrons experience the well-known Lorentz force in the magnetic field, and the electric field force in the electric field, leading to the circular motion of electrons close to the target material, enhancing the collision with the target, and improving the sputtering yield. The sputtering process is accomplished under vacuum to avoid oxygen or other gas contamination that might cause impurities to form on the substrate surface. The Factors affecting the plasma sputtering process are depicted in Figure 6.

### 3.2. Biomedical Applications of Sputtered Electrospun Polymer-Based Nanofibers

Plasma technology can be used to improve the surface properties of polymers without changing their bulk characters. Plasma-treated polymers have found wide application in diverse fields, such as the automobile, microelectronics, chemical and biomedical industries [121]. Polymer surface properties such as hydrophobicity, roughness, chemical structure, conductivity, etc. can be modified for various applications. Plasma treatment can affect the polymer surfaces through micro-etching, organic contamination, cross-linking, surface chemistry modification, and surface coating with a specific target material [122]. The biomaterials should possess good mechanical and surface characteristics that are appropriate for the biological environment. For instance, for cell adhesion, the polymer surface should have low surface free energy, surface roughness, and hydrophilicity. Plasma treatment via magnetron sputtering technology has been implemented to coat the surfaces of polymers to form biomaterials suitable for biomedical applications such as antibacterial, biocompatibility, and tissue engineering.

Plasma sputtering technology includes both thermal and non-thermal deposition processes. However, non-thermal deposition processes are highly recommended for polymers, because they do not damage the bulk properties of the polymer. Magnetron sputtering is the technique used for coating the polymer surface. Magnetron sputtering is a technology that was developed during the 1970s, and it is a high-speed and low-temperature technique for preparing a strong and uniform adhesion film on the surface of polymers, ceramics and composite materials [123]. However, argon gas is commonly used, because it does not damage the target due to its nobility. The full process is quick and requires only a low temperature, while offering a high film forming rate and strong film adhesion [124]. For instance, composite microfibers of Poly(methyl methacrylate)/organically modified montmorillonite (O-MMT) were manufactured by electrospinning with the incorporation of emulsion polymerization [125]. Here, the prepared composite microfibers of PMMA-O-MMT were magnetron sputter-coated with Titanium dioxide (TiO_2_). The results showed that the deposited anatase-TiO_2_ and rutile-TiO_2_ exhibited better surface wettability without damaging the PMMA-O-MMT compound. These composite fibers have a UV absorption of 254 nm. Therefore, it induces the photocatalytic degradation of the model compound methylene blue. Thus, these materials provide a promising application in dye wastewater treatment.

Polymer microspheres [126], thin films [127], and fibers [128] have been coated with Ag [107,108,129], Cu [130], Ti [131], TiO_2_ [132], gold (Au) [133], hydroxyapatite (HAP), tricalcium phosphate (TCP) [134], amorphous calcium pyrophosphate (CPP) [134], and dicalcium phosphate dihydrate (DCPD) [134] for different biomedical applications.

#### 3.2.1. Antibacterial Coatings

The attachment of bacteria to the surface of a polymer can lead to the formation of biofilm. Therefore, biofilm-resistant polymers are an essential factor for the medical field. Biofilm resistance could be imbued in the polymer through the addition of antibacterial agents on the surface of the polymer to prevent bacterial adhesion. Materials such as medical textiles, wound dressings, prostheses and implant materials should display antibacterial activity for efficient biological activity. Antibacterial properties are an essential parameter to take into account for wound dressing. Antibacterial activities are promoted through the addition of some antibacterial components to the fabrics. There are many components, including both inorganic and organic (drugs), as well as metals. Inorganic agents include TiO_2_, carbon nanotubes, and Ag, Zn, ZnO_2_, Cu, Ga, and Au NPs [135]. Organic agents such as Triclosan inhibit the development of micro-organisms using electrochemical activity to disrupt their cell walls [136].

Among the inorganic antibacterial components, Ag NPs have been well studied [137]. Ag NPs were added to electrospun fibers via Ag ions through the wetting process [138,139], silver sulfaazide [93], etc. The wetting process for the addition of Ag to the matrix has many disadvantages, such as uneven distribution of NPs, use of reducing agents that are toxic, and the difficulty of controlling the size of NPs—depending on the strong and weak reducing agents used [140]. However, the most efficient way of introducing NPs to the surface of polymers or fabrics is by using plasma technology. Plasma technology provides more uniform deposition, less use of resources, and a simpler process for the coating of antibacterial material such as Ag, Si, Cu, etc., onto the surface of the polymer than the wetting process. Sputtering, known as physical vapor deposition, has been used effectively in the coating of a number of thin films for electronics applications. Therefore, sputter-coating of metals to enhance antibacterial properties can be performed with the addition of many target materials, including Ag, Ag/Si, Cu, Ti, etc. Therefore, more studies are required to compare the antibacterial properties of various materials incorporated in bioresorbable polymers.

##### Silver (Ag)

Silver is a transition metal in the periodic table. Silver-related compounds or NPs have a biocidal effect on around 16 species of bacteria, because of its toxic effect on microorganisms [119,120,141,142]. Thus, silver is coated on medical devices for antibacterial applications [143]. At low concentrations, Ag NPs show good antibacterial efficiency [144]. Moreover, the lack of toxic effect of Ag NPs on human monocytes cell lines indicates the possible application of Ag in the fabrication of medical devices.

The mechanism of antibacterial activity of Ag on microorganisms has not yet been well studied. It has been shown that, in *E. coli*, AgNP-treated bacteria exhibit some pits on the cell wall and an accumulation of Ag in the cellular membrane. This type of membrane exhibits an increase in permeability. Bacterial DNA loses its replication ability and cellular proteins and becomes denatured by binding Ag ions or NPs to the functional group of the protein [145]. On the other hand, some authors have reported that Ag NPs would denature the cellular proteins required for cellular nutrient transport and damage the cell membrane or cell wall, enhancing cell permeability and ultimately leading to cell death [142]. It has also been noted that the antibacterial efficiency of Ag depends on its shape. Ag NPs with a {111} lattice basal plane (representing the cubic structure lattice pattern) display more robust antibacterial action than spherical and rod-shaped NPs and silver ions [146].

Silver has excellent antibacterial properties. Silver treatment is well known for its application in wound dressing materials. The silver is incorporated as Ag NPs by introduction through AgNO_3_ using a reducing agent. However, this type of silver incorporation leads to burst release of silver from the material, resulting in a very high concentration of Ag in the wound. This is followed by the sudden reduction in silver because of both bacterial consumption and reaction with other compounds present in the wound beds, such as phosphates, chlorine, and proteins. Therefore, silver release in the wound should take place in a controlled manner. Also, the silver nitrate present is a hypotonic; hence, it can cause a strong electrolyte imbalance, which could damage the wound site and produce gross systemic inequality, which could kill patients with extensive burns who require large doses of silver. However, silver sulfaazide was developed in order to minimize the side effects of using silver nitrate. However, the removal of silver sulfaazide cream from the wound surface is performed by scraping, and this could result in a highly painful dressing procedure for the patients. Moreover, sulfaazide does not show any hypotonic effect. Therefore, it is necessary to develop a better process for delivering silver that is efficient, involves introduction over a prolonged period, acts against many ranges of bacteria, requires only a few changes of the wound dressing, and never interferes with the wound healing process. With this in mind, sputtering is a new field of surface coating of wound dressing materials for extended release of silver with potent antibacterial properties. The optimization of the sputtering process is an essential criterion for better antibacterial properties.

It is also noted that silver is the best candidate for wound curing applications, because it reduces inflammation [147], impedes contraction, and improves cell epithelialization [148]. Ag NPs exhibit cellular toxicity, and this leads to a decrease the biocompatibility of the scaffolds [149]. However, the amount of Ag in the scaffolds can be used to optimize the antibacterial effect and cellular toxicity of the Ag.

Antibacterial coatings on medical textiles are an important tool for avoiding infections during the surgical process [150]. Silver is coated onto textiles via different techniques. Silver-coated textiles are limited in application because of their reduced durability. Therefore, strong adhesion of silver on fabrics can be obtained by using the sputter-coating technique [150]. Cotton fabric with antibacterial properties could have a variety of applications. Silver is the most commonly used material for enhancing the antibacterial properties of cellulosic fibers [150]. Wet and dry methods can be used to incorporate silver particles. The wet method changes the bulk properties of textiles and also has a negative impact on the environment. However, dry processes such as sputter-coating are eco-friendly processes than only change the surface of the matrix. Therefore, Ag was incorporated into cotton matrixes of various thicknesses in order to study the antibacterial properties, the release of the Ag in water, etc. The results suggested that Ag shows antibacterial activity against *Staphylococcus aureus*, *Escherichia coli* and *Candida albicans* [150]. In addition, sputter-coating also improved the water contact angle of the cotton fabrics. Thus, antibacterial properties could be added to the nanofibers using the sputtering technique. Simultaneously, Chen et al. sputtered PET fabrics using high-power impulse magnetron sputtering, which provides a highly concentrated plasma, so that these fabrics will support adhered films [151]. Examination of antimicrobial activity revealed that a silver film that is deposited for more than 1 min displays excellent bactericidal (>0) and bacteriostatic (>2.0) effects, based on JIS standards. Furthermore, the coated fabrics showed the capacity to retain antibacterial properties over 20 cycles of washing, indicating the long-term durability for the materials.

**Figure 7:**
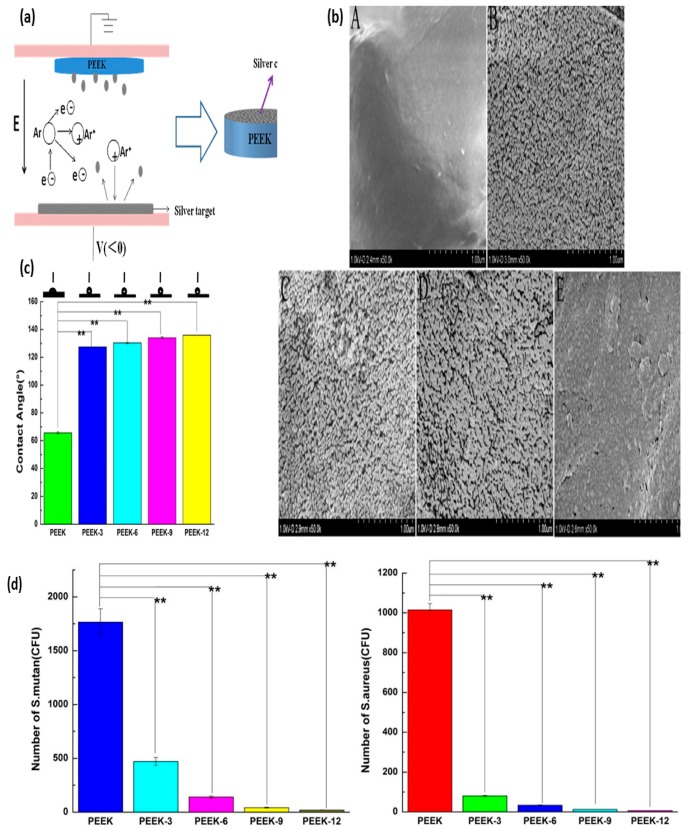
(**a**) Schematic representation of PEEK film coated with nanosilver via sputter-coating; (**b**) SEM images of PEEK at different thicknesses of Ag (3, 6, 9, 12 nm); (**c**) Water contact angle of the coated thin film; (**d**) Antibacterial activity of the PEEK/Ag composite material. Reproduced with permission from [152]. Elsevier, 2017.

The wound dressing mats of polymer sheet and electrospun fiber scaffolds were sputter-coated with Ag and their antibacterial properties studied. Liu et al. examined the influence of magnetron sputter-coating of nanosilver on polyetheretherketone (PEEK) and investigated the resulting cytotoxicity and antibacterial properties [152]. PEEKs were sputter-coated with Ag 3 nm, 6 nm, 9 nm and 12 nm NPs (Figure 7). The antibacterial properties and bacterial adhesion to the surface were studied. Homogeneous nanosilver was coated on the surface; an increase in the water contact angle was observed, and there was no cytotoxicity for the CCK-8. In addition, the coating also provided excellent adhesion of bacteria to the PEEK and improved antibacterial activity towards *Streptococcus mutans* and *Staphylococcus aureus*.

The combination of electrospinning and sputtering technology can result in many novel composite fibers with diverse applications in the biomedical field, such as for wound dressing mats with excellent biocompatibility and antibacterial properties. The electrospun microfibers were coated with Ag by DC magnetron sputtering [129]. The electrospun scaffolds of poly(glycerol sebacate)/poly(3-caprolactone) (PGS/PCL) were coated with Ag, and their antibacterial properties and silver release behavior were studied. PGS/PCL showed good mechanical and thermal behavior due to the increase in fiber diameter and the decrease in fiber pore size when sputter-coated with Ag. The fiber scaffolds demonstrated a gradual release of Ag, contributing to antibacterial activity. Therefore, this material could find appropriate application in wound dressing and bandages. Moreover, prosthetic implants also require antibacterial properties in order to avoid infection after surgery. With the objective of avoiding abdominal infections after implanting prostheses for hernia repair, Muzio et al. [153] prepared polypropylene prostheses coated with a silver-silica composite (Ag/SiO_2_) layer. The prepared mesh hernia prostheses (CMC) consisted of two layers of microporous light mesh and a thin transparent film of polypropylene. The Ag/SiO_2_ composite was sputter-coated onto the CMC meshes and the microporous mesh layer alone. The sputtering process was optimized via addition in order to test biocompatibility and antibacterial properties. In addition, it is noted that sputter-coating with CMC improved the antibacterial properties, but reduced biocompatibility. However, the sputter-coated meshes alone showed good antibacterial properties and biocompatibility. In addition, fiber meshes coated with Ag/SiO_2_ enhanced the growth of seeded fibroblast without causing apoptosis or necrosis of the fibroblast; in addition, the meshes also exhibited good antibacterial properties.

##### Copper (Cu)

In addition to Ag, electrospun scaffolds have been sputter-coated with Cu to improve their antibacterial properties [132,133]. Cu is cheaper than Ag; therefore, Cu coating can provide economical wound dressing mats [154,155]. A PLA scaffold was DC magnetron sputter-coated with copper (Cu) [130]. The PLLA scaffold had increased hydrophobicity, proportional to plasma treatment time. Antibacterial testing concluded that the modified composite scaffold had a bacteriostatic effect in which bacteria were reduced by 30% and 50% [130]. In addition, it was also found that copper has a stronger antibacterial impact than copper oxide. Therefore, this type of composite material could be used for economical wound dressing mats with antibacterial effect. 

*Eichornia crassipes*, commonly known as water hyacinth, is a natural fiber that has found significant applications in recent years [156]. It was sputter-coated with copper (Cu) to study the antibacterial properties. The results revealed that the Cu-coated fibers showed better bacterial inhibition towards *Escherichia coli* (*E. coli*) and *Staphylococcus aureus* (*S. aureus*) compared to pure water hyacinth fibers. In addition, the incorporation of the Cu coating improved the hydrophobicity of the fiber, thereby enhancing the antibacterial activity. 

##### Titanium Dioxide (TiO_2_)

Electrospun chitosan (Cs) nanofibers were sputtered with TiO_2_ particles by plasma-enhanced chemical vapor deposition using a planar magnetron device [132]. The physiochemical interaction of the Cs and Cs-TiO_2_ was studied computationally using the Gaussian software package, followed by experimentally examining the antibacterial properties of the materials. The results revealed that Cs-TiO_2_ behaves as a single composite unit by forming a C-O-C glycosidic bond in the glucose ring. The Cs-TiO_2_ composite had an improved structure and reactivity because of the reduced HUMO-LUMO energy gap, larger dipole moment, and lower ionization potential when compared to pure Cs. Finally, the Cs-TiO_2_ composites exhibited antibacterial activity, with inhibition zones approximately 11.5 mm in diameter.

#### 3.2.2. Surface Modification for Enhancing Biocompatibility

Surface modification via coating can improve the cell adhesion, proliferation, and differentiation; this reduces the risk of thrombosis and imparts bactericidal properties to the stent. In the literature, it has been shown that plasma surface treatment of L-PLA and PCL polymers improves the surface roughness, as well as reducing the surface free energy for better cell attachment of diverse cells to the surface of the polymer mesh [157]. Magnetron-sputtered polymer sheets offer reduced cytotoxicity and better cell viability for biomedical applications. The surface characteristics have been studied for polymer thin films, as well as for electrospun fiber scaffolds. For example, Staszek et al. [158] reported the cytotoxicity of glycerin sputtered with different noble metals such as Au, Ag, palladium (Pd) and platinum (Pt). The results suggested that they had prepared Au, Ag, Pd, and Pt NPs with sizes of 6.1 ± 1.0 nm, 4.2 ± 0.9 nm, 2.5 ± 0.6 nm and 1.9 ± 0.4 nm, respectively. In addition, Pt and Pd demonstrated great cytotoxicity for the 6 cells lines tested (human cells from hepatocarcinoma (HepG2), human keratinocytes (HaCaT), mouse macrophages (RAW264.7), mouse embryonic fibroblasts (L929 and NIH3T3), and cells from Chinese hamster ovary (CHO-K1)), and lower cytotoxicity was noted for Ag and Au after 24, 48 and 72 h.

Consequently, surface modification of hydrophobic polymers via plasma treatment can enhance wettability. E.N. Bolbasov studied the surface modification of PLA and PCL bioresorbable polymers via radio frequency thermal glow discharge plasma using hydroxyapatite as a target in the presence of Ar+ as plasma [127]. The results indicated that the PLA and PCL surfaces showed enhanced biocompatibility for cell line EA-hy926 attachment to the surface. Surface free energy and surface roughness were improved by long exposure to plasma treatment. In addition, plasma sputtering technology can enhance surface roughness, improving cell attachment onto the thin polymers fibers [159]. The PLLA polymer thin film was RF magnetron-sputtered with hydroxyapatite target. This coating led to an increase in biocompatibility with the cells of bone marrow multipotent mesenchymal stromal cells. This was mainly due to the increase in the surface roughness of the PLLA film resulting from the plasma coating, in addition to the enhancement of calcium and phosphorous caused by the hydroxyapatite target. Surface modification of polymer films enhances biocompatibility and reduces cell toxicity.

Furthermore, biodegradable PLA polymer was prepared via electrospinning, and surface modification was implemented by RF magnetron sputtering. The electrospun PLA scaffold was sputter-coated with hydroxyapatite (HAP), tricalcium phosphate (TCP), amorphous calcium pyrophosphate (CPP) and dicalcium phosphate dihydrate (DCPD) [134]. It was found that all prepared fibers showed cytotoxicity because of the production of a toxic compound on the fiber surface, as well as the fact that the fiber surface had been devastated due to the extended plasma treatment.

**Figure 8:**
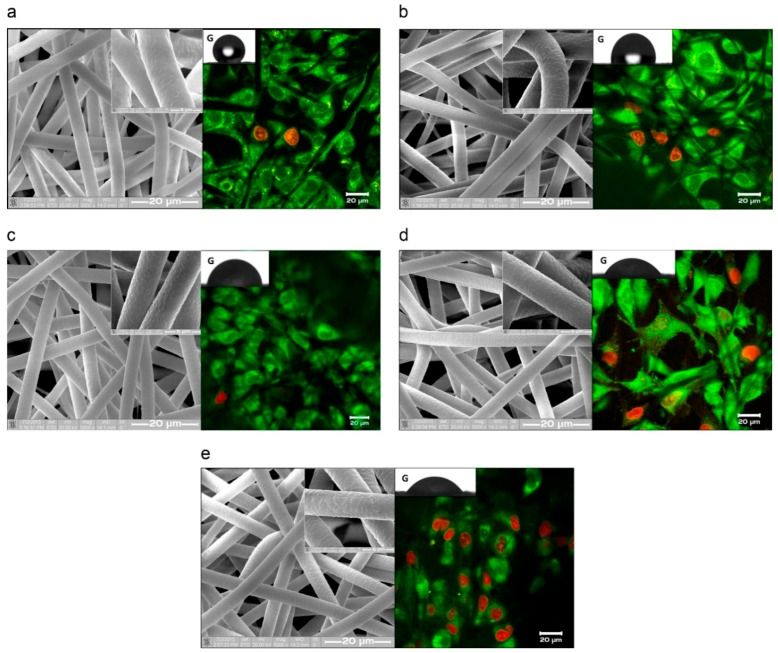
SEM images, fluorescent map of labeled cells (live green cells (acridine orange), orange nucleus of dead cells (ethidium bromide) and wettability for PCL samples that were (**a**) untreated; and treated in plasma for (**b**) 1 min; (**c**) 3 min; (**d**) 6 min; and (**e**) 9 min. Reproduced with permission from [131]. Elsevier, 2016.

On the other hand, the same team of scientists worked on PCL scaffold fibers that were magnetron sputter-coated with titanium targets (Figure 8) [131]. They found that hydrophilicity improved with an increase in plasma treatment time. In addition, increasing the number of pores on the fiber structure did not affect the mean fiber diameter. As plasma treatment time was increased, the adhesion of cells improved. Consequently, cell viability decreased when plasma treatment time reached 9 min.

In another study, poly (l-lactic) acid (PLLA) scaffold was sputter-coated with titanium target under a nitrogen atmosphere [160]. The pure PLLA did not show any changes in its physiomechanical properties. Biocompatibility testing in in vivo rat models indicated that there was no severe tissue reaction after around three months for the implemented subcutaneous tissue. Finally, the replacement of scaffolds from the recipient tissue depends on plasma treatment time.

#### 3.2.3. Tissue Engineering

Plasma technology has also emerged recently for use in tissue engineering applications such as vascular grafting, stem cell therapy, and artificial muscle sputter-coated with conductive Au. For this purpose, highly biocompatible polymers have provided a platform for cell adhesion, proliferation, and differentiation. To this end, stem cells are added to the polymer scaffold to provide a better tissue regeneration environment. Stem cell therapy is a new platform that may act as an alternative to many complicated surgical procedures. Stems cell-loaded materials have gained much attention recently [126]. Lee et al. [126] reported the use of PCL microspheres sputter-coated with Au as a platform for differentiating cardiomyogenic cells from human embryonic stem cells. They sputtered the PCL microspheres for 5 min, and then incorporated the human embryonic stem cells (hESCs). It was noted that these composites showed a higher cardiac differentiation, because Au acted as the mediator for gene expression on day 4 and day 14 [126].

Moreover, PLLA and PEG fibers were electrospun and sputter-coated with calcium phosphate for bone tissue engineering applications. Here, simple combination of the electrospinning and sputtering techniques is feasible for the fabrication of biopolymer scaffolds for biomedical applications [161].

Many novel composite materials have emerged due to the fusion of two valuable techniques, such as the electrospinning and sputtering techniques [162]. Innovative materials have been studied for vascular tissue engineering. These composite materials were prepared using the electrospinning and sputtering technique. To this end, PCL and PHBV were incorporated at a ratio of 1:2 (*v*/*v*) and sputter-coated with Ti. Firstly, the sputtering process was optimized so that it would not damage the macrostructure of the scaffolds. The biocompatibility of the prepared composite mats was studied with hybridoma of the endothelial cells of the human umbilical vein and human lung carcinoma (EA.hy.926 cell line). The results showed that cell adhesion was improved for Ti-coated scaffolds, and that they exhibited better proangiogenic activity.

**Figure 9:**
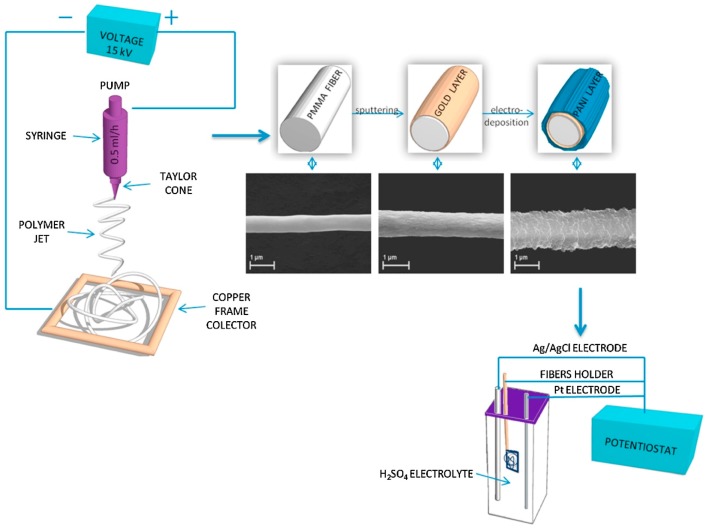
Schematic representation of electrospinning and sputtering for muscle tissue engineering applications. Reproduced with permission from [133]. Elsevier, 2016.

Furthermore, a novel approach was applied to process fibrous scaffolds for artificial muscles or human body smart devices by combining electrospinning and sputtering technologies [133]. A core/shell structure was made by first electrospinning the PMMA in optimized form to obtain a uniform fiber; later, the PMMA was coated with Au to induce conductivity and obtain suitable mechanical properties in the scaffolds. Subsequently, polyaniline (PANI) was coated onto the scaffolds via in situ electrochemical polymerization, starting with aniline and using sulfuric acid as an oxidizing agent (Figure 9). PANI-coated metalized fiber scaffolds in a structure similar to the core/shell structure showed fascinating electrochromic properties, in which color changes occurred when the applied voltage was switched from 0 to 1 V, and vice versa. In vitro biocompatibility testing revealed good cell adhesion, with a better result shown when tested on human amniotic fluid stem cells than on eukaryotic cells. Therefore, this type of web could be used to prepare smart artificial muscle devices via a versatile and straightforward preparation technique using electrospinning and sputtering.

## 4. Conclusions

Throughout this review, new insights for biomedical applications have been addressed, focusing predominantly on the promising benefits of employing sputtered electrospun polymer-based nanofibers. It is evident from the number of ineffective conventional treatments that there is a desperate necessity for distinct and unique therapies. As addressed extensively in this paper, combining sputtering and electrospinning technologies has the potential to play a critical function in different biomedical applications such as antibacterial coatings, surface modification for enhancing biocompatibility, and tissue engineering. Investigated by means of various nanostructure studies, the above-mentioned concepts can be used in an attempt to strengthen overall therapeutic behavior. As represented through the numerous reports addressed, therapies including silver and copper nanoparticles have the potential to be applied directly in different biomedical applications. Overall, it is clear that the field of combined sputtering and electrospinning technologies is progressing at an incredibly fast rate, providing promising behaviors for various biomedical applications.
